# A Comparative Evaluation of the Genotoxic Effects of Mobile Phone Radiation Using Buccal Micronucleus Assay

**DOI:** 10.30476/dentjods.2022.92515.1656

**Published:** 2023-03

**Authors:** Hamideh Kadeh, Shirin Saravani, Mahsa Moradi, Niloofar Alimanesh

**Affiliations:** 1 Oral and Dental Disease Research Center, Dept. of Oral & Maxillofacial Pathology, School of Dentistry, Zahedan University of Medical Sciences, Zahedan, Iran; 2 Postgraduate Student, Dept. of Pediatric Dentistry, School of Dentistry, Zahedan University of Medical Sciences, Zahedan, Iran; 3 Dentist, School of Dentistry, Zahedan University of Medical Science, Zahedan, Iran

**Keywords:** Cell Phone, Buccal Mucosa, Micronuclei, Genotoxicity

## Abstract

**Statement of the Problem:**

Mobile usage has increased worldwide over the past two decades. There are conflicting reports about the carcinogenic effects of cell phone radiation on the oral mucosa. Micronucleus (MN) is considered a reliable marker for genotoxic damage.

**Purpose:**

This study aimed to identify the impact of mobile phone radiation on the MN frequency in oral mucosal cells.

**Materials and Method:**

In this descriptive-analytical study, 50 mobile phone users between the age group of 20–38 years were included. Samples were obtained from the right and left cheek mucosa of each subject (a total 100 cell samples). Every participant filled out a questionnaire about his or her cell phone usage habits. Additionally, personal information such as age, gender, and body mass index (BMI) were assessed. The Feulgen and Papanicolaou staining methods were used for staining of the cell samples. A total of 1000 cells in each sample were evaluated for MNs.

**Results:**

The mean number of MN in exposed and non-exposed mucosa by Feulgen method was 0.71±1.13 and 0.57±1.36, respectively. Also in Papanicolaou staining, the mean number of MN in the exposed mucosa and non-exposed mucosa was 6.94±6.61 and 6.54±6.88, respectively, but these differences were not significant (*p*> 0.05). The frequency of MN in non-specific DNA staining was significantly (5- to 6-fold) higher than DNA-specific staining. We observed no statically significant differences between MN frequency according to age, gender, BMI, and other cell phone usage habits (*p*> 0.05).

**Conclusion:**

This study showed that cell phone use does not cause genotoxic effects in the buccal mucosa in the oral cavity. Moreover, using non-specific DNA staining methods can increase the frequency of MN by more than 5- to 6-fold.

## Introduction

Over the past two decades, the use of mobile phones has become almost universal [ 1 ]. The number of mobile phone users reached 4.93 billion in 2018 and increased to 5 billion in 2019 [ 2 ]. Radiofrequency radiation (RFR) is a type of electromagnetic radiation that varies from 3 kHz to 300 GHz. Most cell phones transmit RFR signals within the 800-900 and 1,800-2,200 MHz ranges [ 1 ].

The global growth of mobile phone usage has risen concerns about the dangers of electromagnetic waves associated with this technology [ 3 ]. There are two separate perspectives on the impact of radio waves on health, the first of which is due to the long conversations raising the heat of cell phones and the temperature of contact with the surrounding tissues. The second reason may be the non-thermal effects of waves from mobile phones and transmitter stations [ 4 ].

The biological consequences of exposure to radio frequencies are controversial [ 5 ]. Decades of research have yielded conflicting results; for instance, the results of some studies were reported the existence of a causal relationship between exposure to radiofrequency electromagnetic fields with an increased risk of glioma, meningioma, infertility, and deafness [ 6 - 9 ]. However, the findings of other studies could not confirm these results [ 10 - 12 ]. 

Micronuclei (MNs) are structures that contain chromosome fragments or whole chromosomes that are not incorporated into the nucleus of a daughter cell during cell division due to failure to bind to the spindle, and therefore, reflect aneugenic or clastogenic damage [ 13 ]. According to Bonassi *et al*. [ 14 ], they are considered as markers for the risk of cancer. The micronucleus (MN) test is one of the most common methods used to evaluate exposure to mutagens, carcinogens, and DNA damage [ 14 - 17 ]. It determines the presence of small nuclear bodies called micronuclei. The abundance of micronuclei in peripheral blood lymphocytes is associated with the risk of cancer and cardiovascular disease, making it a reliable marker [ 14 , 18 ]. 

MN can also be counted in exfoliated cells, especially in the oral mucosa [ 15 ]. The use of MN assay of buccal mucosa cells has various advantages, such as highly fast and convenient cell extraction. Moreover, it is especially suitable for determining the effects of RFR emitted by mobile phones because the oral cavity is in the exposure area and there is a possibility of genotoxic changes in this area [ 3 , 15 ]. Current reports about the effect of cell phones radiation on nuclear anomalies in oral mucosal cells are contradictory [ 3 , 15 - 16 , 19 ]. Therefore, this study aimed to investigate the effects of exposure to cell phones radiation on the MN frequency in the epithelial cells of buccal mucosa in the oral cavity.

## Materials and Method

### Subjects

This research was approved by the Ethics Committee of Zahedan University of Medical Sciences, Zahedan, Iran, (IR.ZAUMS.REC.1397.368) (Project No.9082 and 6776). The participants of this study consisted of 50 individuals who were explained about the purpose of this project and obtained informed consent. Afterward, a pre-designed questionnaire was filled out for each patient that included patients' demographic information, such as age, gender, and body mass index (BMI). Also, information about their cell phone usage habits such as usage duration within the week, years of cell phone use, usage of headphones and preferential side of cell phone usage was retrieved.

The inclusion criteria were defined as individuals in the age limit of 20-40 years, without history of systemic disease, having radiation in the previous 2 months, and occupations in contact with chemicals. The exclusion criteria were defined as smokers, tobacco, and alcohol users and having any oral mucosal lesion. 

### Sample Collection and Staining

We collected two cell samples from the right and left cheeks of each subject, a total of 100 cell samples from the buccal mucosa exposed and buccal mucosa none/ less exposed. The cases that used their cell phones with the same frequency on both sides were excluded from the research. 

According to the method proposed by Thomas *et al*. [ 20 ], the patients were first asked to rinse their mouth with water to remove food particles, debris, and saliva. Subsequently, exfoliated cells of buccal mucosa were collected using a cotton swab and applying circular motion 20 times. A separate swab was used for each cheek. The samples were spread on a glass slide, and then the prepared smears were fixed in Carnoy’s solution for 30-35 min and finally were stained by DNA-specific (Feulgen) and non-specific DNA (Papanicolaou) staining according to the manufacturer’s instructions.

### Evaluation of Micronuclei

The samples were then counted by a pathologist for the number of MN under an optical microscope (Nikon, Japan). The MN count was determined by the number of counted MN per 1000 cells (cells with a clear margin and nucleus were considered not overlapped cells) per subject using the at 400×magnification. Mean number of MN were counted for all samples and were presented as mean±SD. The pathologist was blind to the information of the subjects. The criteria introduced by Tolbert *et al*. [ 21 - 22 ] were used to detect MN as (1) a clear, smooth, and round perimeter suggesting a membrane, (2) nuclei with a third the diameter of the associated nucleus but with a specific color and shape, (3) staining intensity similar to the nucleus, (4) texture similar to the nucleus, and (5) no overlap or bridge with nucleus.

### Statistical Analysis

The data were analyzed in SPSS version 21 (SPSS Inc, Chicago, IL) using the paired t-test, independent samples test, one-way Anova and Pearson correlation coefficient. p Value less than 0.05 was considered statistically significant.

## Results

In the present study, collecting buccal mucosa cells from the right and left cheeks of 50 mobile phone users (a total 100 samples) were examined to determine the MN frequency using two staining methods. The subjects were in the age range of 20-38 years with a mean age of 25.13.87 years and 52% of them were males. It was revealed that most of the subjects (80%) used their right cheek more frequently for a cell phone conversation, which was considered as exposed mucosa, while in 20% of cases, who used their left cheek more often during conversations, the buccal mucosa of the left side was considered as the exposed mucosa. The subjects who used both cheeks with the same frequency to talk on cell phones were excluded from the study. Other details about the subjects are given in Table 1. 

**Table 1:** Characteristics of the study population

Characteristics		Frequency N (%)
Sex	Male	26(52)
	Female	24(48)
Body Mass Index (BMI)	<20	9(18)
	20-25	32(64)
	>25	9(18)
Overall period of exposure	<5 years	2(4)
	5-10 years	30(60)
	>10 years	18(36)
Duration of phone use (h/ week)	˂1	5(10)
	1-5	28(56)
	>5	17(34)
Side of the face in which the mobile phone is placed	Right	40(80)
	left	10(20)
Headset usage	Yes	20(40)
	No	30(60)

**Figure 1:**
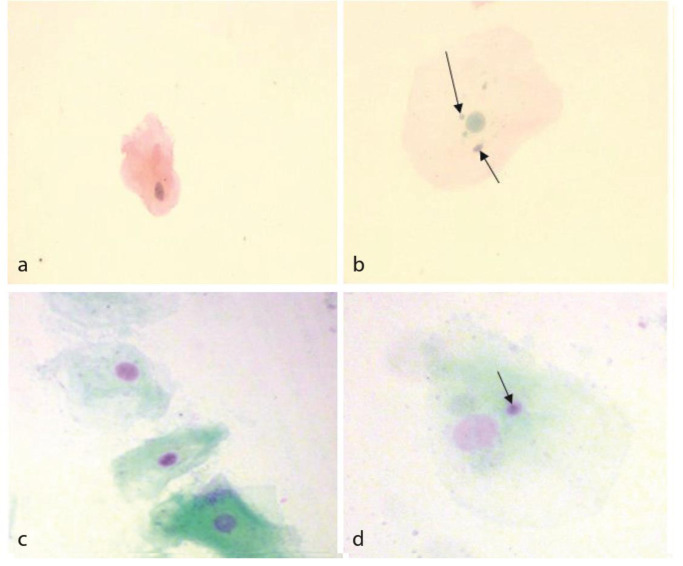
**a:** A single cell with a normal nuclei (400×) using Papanicolaou staining and **b:** A single cell containing multiple micronuclei (400×) using Papanicolaou staining, **c:** Cells with normal nuclei (400×) using Feulgen staining and **d:** A single cell containing a micro-nuclei (400×) using Feulgen staining

The mean numbers of MN in exposed and non-exposed mucosa by Feulgen method were 0.71±1.13 and 0.57±1.36, respectively; the difference was not statistically significant (Table 2) (Figure 1). Also in Papanicolaou staining, the mean number of MN in the exposed mucosa and non-exposed mucosa was 6.94±6.61 and 6.54± 6.88, respectively, which was not significant (Table 3) (Figure 1).

**Table 2:** Micronucleus count in exposed and non-exposed Buccal mucosa by Feulgen staining

Groups	Micronucleus (Mean±SD)	*p*
Exposed mucosa	0.71±1.13	0.459
Non exposed mucosa	0.57±1.36	

**Table 3:** Micronucleus count in exposed and non-exposed Buccal mucosa by Papanicolaou staining

Groups	Micronucleus (Mean±SD)	*p*
Exposed mucosa	6.94± 6.61	0.285
Non exposed mucosa	6.54±6.88	

It can be seen that the MN frequency were significantly (~ 6-fold) higher in non-specific DNA (Papanicolaou) than DNA-specific (Feulgen) staining. In both staining methods an increase in the mean of MN frequency was observed in men, individual which used mobile phone for more than 5 h per week and more than 10 years, but this difference was not statistically significant (Tables 4-5). Moreover, the mean of MN frequency was lower in subjects using headphones than in those who did not use headphones, which was not significant.

**Table 4:** Mean micronucleus count in relation to characteristics of the samples staining with Feulgen method

Characteristics		Micronucleus Mean±SD	*p*
Sex	Male	0.76± 1.19	0.690
	Female	0.63± 1.06	
Overall period of exposure	<5 years	0	0.109
	5-10 years	0.86± 1.12	
	>10 years	1± 2.0	
Duration of phone use (h/week)	<1	0.50±0.75	0.064
	1-5	0.60±0.94	
	>5	0.66±1.11	
Headset usage	Yes	0.40±0.75	0.126
	No	0.90±1.29	

**Table 5:** Mean micronucleus count in relation to characteristics of the samples staining with Papanicolaou method

Characteristics		Micronucleus Mean±SD	*p*
Sex	Male	7.50± 6.68	0.539
	Female	6.33± 6.63	
Overall period of exposure	<10 years	6.21±5.84	0.309
	>10 years	8.22± 7.82	
Duration of phone use (h/week)	<1	5.20±5.49	0.505
	1-5	6.35±6.21	
	>5	8.41±7.58	
Headset usage	Yes	6.10±6.34	0.469
	No	7.5±6.84	

In the quantitative analysis of some variables (age, BMI, cell phone usage hour/week, and years of cell phone) we observed significant correlation between cell phone usage hours and the number of MN in the case group (r=0.436, *p*= 0.002). Furthermore, a significant correlation was observed between age and the mean of MN frequency in Papanicolaou staining (r= 0.440, *p*= 0.001).

## Discussion

Today, the use of mobile phones is considered an essential part of modern life. One of the biggest concerns regarding this issue is the relationship between cell phone usage and cancer. Although some studies from experimental investigations in animals to extensive epidemiological studies have been conducted, this problem has not yet been resolved [ 2 , 15 ]. 

The oral cavity is located in the area exposed to cell phone radiation, and the epithelial tissue is a target tissue for carcinogenic lesions. On the other hand, the MN test on buccal mucosa is very reliable and widely used since not only can it detect DNA damage but also it is able to evaluate chromosomal instability and cell death [ 15 ]. In addition, it is a reliable marker for an abnormal cell cycle following ectopic mitosis [ 2 ]. This test is a sensitive, non-invasive, low-cost, fast, and easy technique, in which oral mucosal cells similar to different types of cells especially lymphocytes, do not need to be cultured [ 19 ].

In different studies, various wooden spatulas or brushes were utilized to extract and isolate the cells [ 16 , 19 , 23 ]. Nonetheless, in this research, cotton swabs were used since this method is more reliable for cell recovery and more convenient for participants [ 15 ]. In the present study, the mean MN frequency in the exposed and non-exposed buccal mucosa to mobile phone radiation was examined using two staining methods. According to the results of the current study, the mean number of MN in the exposed mucosa of the subjects was not significantly different from that in the non-exposed mucosa, which was consistent with the studies conducted by Hintzsche *et al*. [ 15 ], Ros-Lior *et al*. [ 19 ] Souza *et al*. [ 13 ], and de Oliveira *et al*. [ 15 ].

Vanishree *et al*. [ 2 ] examined two groups of mobile phone users, including a group of mobile phone users less than 5 years with 4-5 hours/week, and a group of mobile phone users over than 5 years with more than 10 hours/week. In their study, Papanicolaou staining was used and a significant increase in the mean of MN was observed between groups, which was inconsistent with the findings of the present study. In Vanishree *et al*. [ 2 ] study, a significant increase was observed in the mean MN among individuals using code division multiple access (CDMA, 1800 MHZ) mobile phones than among those who used the global system for mobiles (GSM, 900 MHZ) mobile phones. They also reported a significant reduction in the mean MN in cases that used headphones; using headphones helps keeping the phone away from the body and subsequently eliminate the direct effect of RF on the body and reduce the local temperature around the ear area [ 2 ]. Finally, they noted that using cell phones, even within an acceptable distance from the body, could cause genotoxicity when used for a long time [ 2 ]. Additionally, when the cell phone is used predominantly on one side of the face, it may cause more genotoxicity due to the increased radiation and heat [ 2 ]. In our study, the mean MN was lower in cases that used headphones than in those who did not use them; however, this difference was not statistically significant. 

In Banerjee *et al*. [ 23 ] study, individuals divided into two groups of the less mobile users (less than 5 years with 3 hours/week) and high mobile users (more than 5 years with 10 hours/week), and determined the number of MN using acridine orange staining. Based on the results of this research, a significant increase of MN mean was reported in the high mobile users group. In addition, the mean MN was significantly lower in cases utilizing headphones. It was also revealed that the subjects who complained more about heat around their ears had a higher mean MN, indicating that heat had a strong synergistic effect possibly by activating heat shock proteins on genotoxic damage [ 23 ].

It has been reported that heat shock protein 70 increases the radioadaptive response [ 24 ]. Furthermore, a local increase in temperature may damage the mitochondrial membrane and lead to the release of cytochrome C and activation of caspases-3 and -9 [ 25 ]. In a study performed by Daroit *et al*. [ 3 ], the effect of cell phones was investigated on the cytogenic abnormalities of oral mucosal cells in different areas (i.e., lower lip, tongue border and mouth floor). Accordingly, a slight increase was observed in the number of micronucleated cells in the lower lip and binucleated cells in the mouth floor among subjects who used mobile phones for more than 60 min per week. It was also mentioned that exposure to cell phone electromagnetic radiation might be associated with the development of nuclear anomalies among individuals using cell phones more than 60 min/ week for more than 8 years [ 3 ]. Yadav and Sharma [ 16 ] compared the mean MNs in 85 mobile phone users and 24 non-mobile users (control group) using the orcein technique. The findings of this study indicated a significant increase in the number of MN among mobile phone users, which was inconsistent with the results of the current study. 

As can be seen, different results have been reported regarding the genotoxic effects of mobile phones on the buccal mucosa, which can be attributed to several reasons. In this regard, one of the important factors leading to discrepancies in the results is related to the used staining method in various studies [ 13 , 15 - 16 , 19 ]. For instance, in a study carried out by Yadav and Sharma [ 16 ], the orcein staining technique was used, which was non-specific for DNA. In this non-specific technique, not only are micronuclei identified but also other artifacts associated with genomic instability may be stained; therefore, an increase in the number of MN can be reported. 

Nonetheless, DNA-specific staining techniques were employed in other studies including researches conducted by Hintzsche and Stopper [ 15 ], Ros-Lior *et al*. [ 19 ], Souza *et al*. [ 13 ] and Daroit *et al*. [ 3 ], in which Chromomycin A3, 4′, 6-diamidino-2-phenylindole, acid-Schiff's reagent, and Feulgen were used, respectively. Nersesyan *et al*. [ 26 ] showed that the use of non-specific DNA staining methods could increase the frequency of MN by more than four times. This finding was similar to our study. In the present study, two methods of staining were used, including DNA-specific (Feulgen) and non-specific DNA (Papanicolaou), resulting in an increase in the number of MN in the non-specific Papanicolaou method. Consequently, it is recommended to employ DNA-specific methods in future studies, which provide added specificity to the results.

Another point that can explain these discrepancies is different sample sizes and also the difference in the number of cells quantified for the assessment of MN. Because the presence of MN rarely occurs, some researchers suggest that at least 1000 cells should be studied, and if less than 5 micronucleated cells are observed after counting 1000 cells, the number of studied cells should be increased to 2000-3000 cells [ 20 , 27 - 28 ]. In our study, 1000 cells were investigated in each sample, which can also be one of the reasons for the discrepancies in the results of the present research with those of other studies.

In this study, we did not find any statistically significant changes in MN frequency regarding age, gender, and BMI. Similar to our study, Hintzsche and Stopper [ 15 ] reported that no significant difference was observed in the frequency of MN considering the factors of gender, BMI, and smoking.

In the current study, the mean of MN frequency was not significantly different in the subjects according to duration of mobile phone use per week, and years of using mobile phones. In a study carried out by de Oliveira *et al*. [ 5 ], in agreement with the results of the present study, the mean MN scores were not significantly different in the subjects in terms of duration of daily cell phone use, and years of cell phone use. 

In the present study, subjects under 40 years were selected for research purposes since aging is considered a risk factor for the frequency of MN [ 19 ]. In this respect, the quantitative analysis of variables in our study showed a significant difference in the relationship between an increase in the age and the mean of MN in the subjects examined using the Papanicolaou staining method. We suggest future studies with larger sample size on this subject. 

## Conclusion

According to the results of the present study, although an increase in the number of MN was observed in the exposed mucosa to mobile radiation compared to non-exposed mucosa, this difference was not statistically significant. The use of non-specific DNA staining (Papanicolaou) methods can increase the frequency of micronuclei by more than six times, therefore employing DNA-specific methods (Feulgen) in future studies is recommended. In addition, no genotoxic effects as a result of exposure to mobile radiation were observed in the oral mucosa in relation to any parameter. 

## Acknowledgement

The authors would like to thank Zahedan University of Medical Sciences for financial support. 

## Conflict of Interests

The authors declare that they have no conflict of interest.
